# Causes of death in patients with autoimmune and rheumatic diseases—a 16-year autopsy-based study

**DOI:** 10.4322/acr.2023.430

**Published:** 2023-05-08

**Authors:** Marta Juliana Mantilla, Juan José Chaves, Juan Camilo Santacruz, Gustavo Rodríguez-Salas, Igor Rueda, Ana Maria Santos, John Londoño, Julio Cesar Mantilla

**Affiliations:** 1 Universidad de La Sabana, Departamento de Reumatología, Chía, Colombia; 2 Fundación Universitaria de Ciencias de la Salud, Departamento de Patología, Bogotá, Colombia; 3 Universidad Industrial de Santander, Departamento de Patología, Bucaramanga, Colombia

**Keywords:** Autoimmune Diseases, Forensic Medicine, Autopsy, Infections, Colombia

## Abstract

**Introduction:**

the autopsy is an essential medical procedure; however, its use has declined over the decades. In autoimmune and rheumatological diseases, anatomical and microscopic diagnosis is critical to diagnose of the cause of death. For this reason, our objective is to describe the cause of death in patients diagnosed with autoimmune and rheumatic diseases who underwent an autopsy in a Pathology reference center in Colombia.

**Materials and methods:**

a retrospective and descriptive study of autopsy reports.

**Results:**

between January 2004 and December 2019, 47 autopsies of patients with autoimmune and rheumatological diseases were performed. Systemic lupus erythematosus and rheumatoid arthritis were the most common diseases. The leading cause of death was related to infections, being opportunistic infections in the majority of the cases.

**Conclusions:**

our autopsy-based study was focused on patients with autoimmune and rheumatological conditions. Infections are the leading cause of death, particularly opportunistic infections, diagnosed mainly by microscopy. Thus, the autopsy should continue to be considered the “gold standard” to determine the cause of death in this population.

## INTRODUCTION

The autopsy is an essential procedure in Medicine since it provides precisely the cause and manner of death.^1^ Despite its multiple benefits (medical care, medical education, research, and public health), its performance in medical centers has declined from rates of 50% in the 1960s to around 4-8% currently.^2-4^ In Colombia (South America), general studies have been conducted describing the main demographic characteristics of clinical autopsies with the respective analysis of the leading causes of death.^5-7^

Autoimmune diseases (AD) and rheumatologic diseases (RD) are heterogeneous groups characterized by immune dysregulation leading to inflammation and tissue damage with multi-organ involvement.^8^ Its prevalence worldwide is around 4-5%.^9^ Mortality rates associated with AD and RD activity are generally low, being the causes of death associated with acute events, such as infections, respiratory diseases, and cardiovascular events.^10,11^ However, studies have shown that patients with AD and RD have a lower life expectancy and increased mortality compared to the general population.^12,13^ Some reports indicate a discrepancy between clinical and post-mortem causes of death diagnosis.^14,15^

The discrepancy in the cause of death between the clinical diagnosis and the pathologist report is around 20%; the microscopic examination provides 5% of the leading causes of death.^16^ The autopsy is considered the “gold standard” for determining the cause of death.^17^ This study aims to determine the cause of death in patients diagnosed with autoimmune and rheumatic diseases who underwent a clinical autopsy in a forensic pathology reference center in Colombia.

## MATERIALS AND METHODS

A retrospective and descriptive study was carried out using the database of the Pathology Department at Universidad Industrial de Santander (UIS) in Bucaramanga, Colombia. A total of 4430 autopsies were performed between January 2004 and December 2019 in patients whose death occurred at Hospital Universitario de Santander (HUS) or other hospitals in the metropolitan area. The study was conducted according to the Declaration of Helsinki guidelines and Colombia's legal regulations.

**Figure 1:**
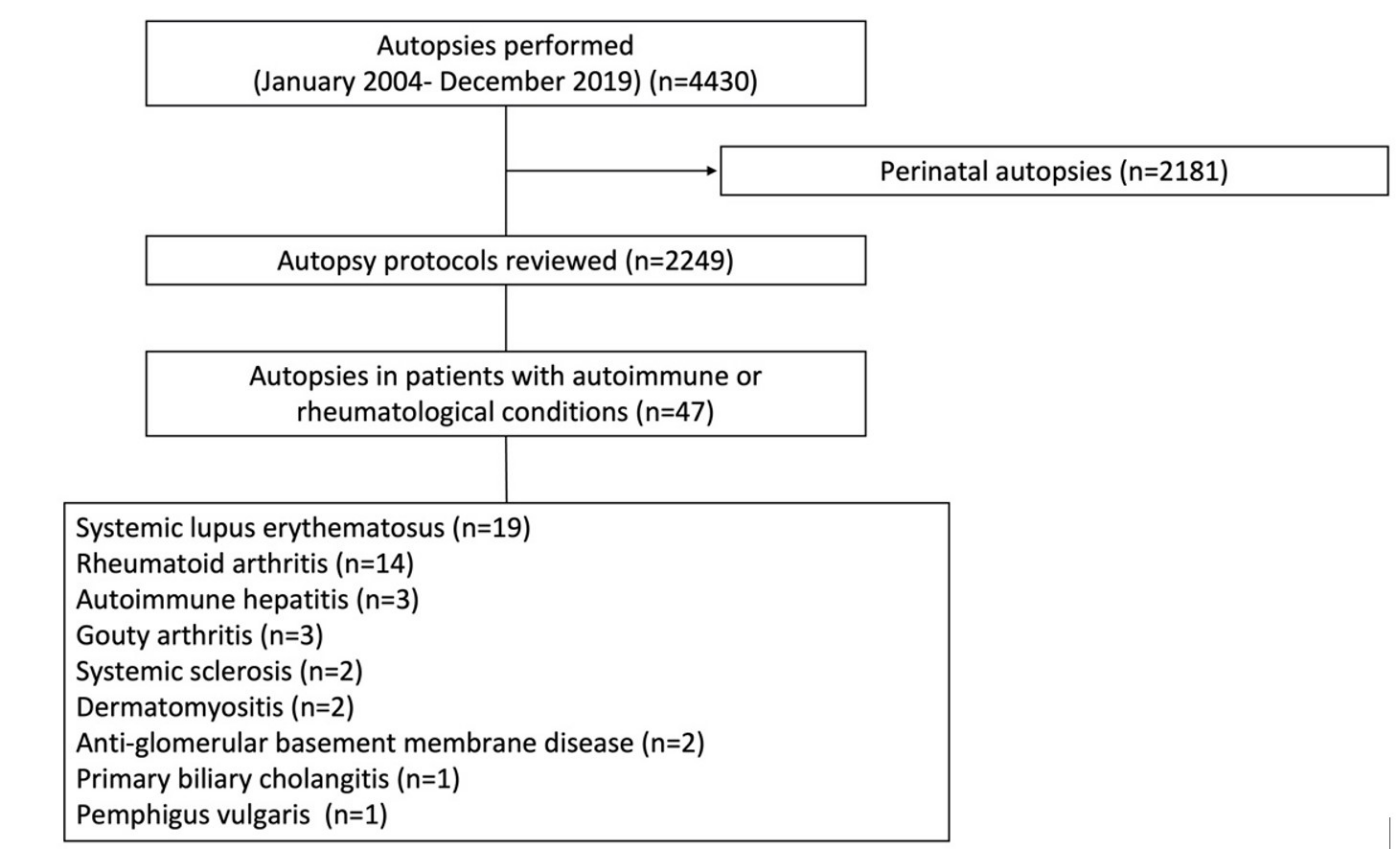
Flowchart of case selection.

Autopsies were performed on patients diagnosed with autoimmune or rheumatic disease who died without a clear cause of death or had a clinical diagnostic impression during hospitalization but died despite the received treatment. In addition, the family had to authorize the autopsy to be carried out. Perinatal autopsies were excluded. After applying these criteria, 2249 autopsy protocols were analyzed, of which 47 corresponded to patients with autoimmune or rheumatological conditions (Figure 1).

## RESULTS

47 cases were included in the analysis, of which 27 (57.5%) were female and 20 (42.5%) were male. The mean age was 39 years old, with a range from 13 to 69. The most common diseases were systemic lupus erythematosus (SLE) (n=19, 40.42%) and rheumatoid arthritis (RA) (n=14, 29.78%), followed by autoimmune hepatitis (AH) (n=3, 6.38%), gouty arthritis (GA) (n=3, 6.38%), systemic sclerosis (SSc) (n=2, 4.26%), dermatomyositis (DMM) (n=2, 4.26%), anti-glomerular basement membrane (anti-GBM) disease (n=2, 4.26%), primary biliary cholangitis (PBC) (n=1, 2.12%), and pemphigus vulgaris (PV) (n=1, 2.12%).

Infections were the most common cause of death at autopsy (opportunistic infections, infection due to common microorganisms, and septic shock) (n=31, 65.96%). Opportunistic infections (OI) were the highest prevalent cause of death (n=17, 37.17%). Tuberculosis was found in 12 cases (25.53%), while further OI or co-infections were cryptococcosis (n=3, 6.38%), systemic candidiasis (n=2, 4.26%), pneumocystosis (n=2, 4.26%), histoplasmosis (n=2, 4.26%), toxoplasmosis (n=2, 4.26%), and aspergillosis (n=1, 2.12%). Other microorganisms described were Cytomegalovirus (CMV) (n=1, 2.12%) and Varicella-zoster virus (VZV) (n=1, 2.12%).

The organic compromise was the second cause of death in AD or RD patients. In these patients, infectious involvement was previously ruled out. 5 (10.64%) patients had renal failure, 3 (6.38%) hepatic failure, and 3 (6.38%) multiple organ dysfunction syndrome (MODS). Lastly, myocardial infarction was reported in 3 (6.38%) patients (2 with RA and 1 with GA), while pulmonary embolism was described in two patients (4.26%) (one in SLE and the other with RA). There is no report of antiphospholipid syndrome in patients with pulmonary embolism and myocardial infarction. A detailed description of the case series is displayed in Table 1.

**Table 1:** Cause of death in different autoimmune or rheumatological conditions, n, (%)

Cause of death	SLE	RA	AH	GA	SSc	DMM	Anti-GBM disease	PBC	PV	Total
Opportunistic infection	5 10.64%	6 12.76%	-	2 4.26%	1 2.12%	2 4.26%	-	1 2.12%	-	17 37.17%
Pneumonia	5 10.64%	3	1 2.12%	-	1 2.12%	-	-	-	-	10 21.27%
		6.38%								
Renal failure	3	-	-	-	-	-	2 4.26%	-	-	5 10.64%
	6.38%									
Septic shock	2	2	-	-	-	-	-	-	-	4
	4.26%	4.26%								8.51%
Hepatic failure	-	-	2 4.26%	-	-	-	-	-	1 2.12%	3
										6.38%
MODS	3	-	-	-	-	-	-	-	-	3
	6.38%									6.38%
	
MI	-	2	-	1 2.12%	-	-	-	-	-	3
		4.26%								6.38%
PE	1	1	-	-	-	-	-	-	-	2
	2.12%	2.12%								4.26%
Total	19 40.42%	14 29.78%	3 6.38%	3 6.38%	2 4.26%	2 4.26%	2 4.26%	1 2.12%	1 2.12%	47 100%

AH = autoimmune hepatitis; anti-GBM = anti-glomerular basement membrane disease; DMM = dermatomyositis; GA = gouty arthritis; MI = myocardial infarction; MODS = multiple organ dysfunction syndrome; PE = pulmonary embolism; PBC = primary biliary cholangitis; PV = pemphigus vulgaris; RA = rheumatoid arthritis; SLE = systemic lupus erythematosus; SSc = systemic sclerosis.

After analyzing the clinical history received before the autopsy, it was possible to conclude that most patients had had a prescription for immunosuppressive therapy; however, treatment adherence is unclear. The most used agents were prednisone (87.23%), methotrexate (19.14%), azathioprine (14.89%), cyclophosphamide (4.26%), and TNF inhibitors (4.26%) (Table 2).

**Table 2:** Immunosuppressive medication use

Medication	SLE	RA	AH	GA	SSc	DMM	Anti-GBM disease	PBC	PV	TOTAL
Prednisone	18	11	2	3	2	2	2	-	1	41 (87.23%)
Methotrexate	-	9	-	-	-	-	-	-	-	9 (19.14%)
Azathioprine	3	-	2	-	1	-	-	-	1	7 (14.89%)
Cyclophosphamide	2	-	-	-	-	-	-	-	-	2 (4.26%)
TNF inhibitors	-	2	-	-	-	-	-	-	-	2 (4.26%)

anti-GBM = anti-glomerular basement membrane disease; AH = autoimmune hepatitis; DMM = dermatomyositis; GA = gouty arthritis; PBC = primary biliary cholangitis; PV = pemphigus vulgaris; RA = rheumatoid arthritis; SLE = systemic lupus erythematosus; SSc = systemic sclerosis; TNF = tumor necrosis factor.

## DISCUSSION

AD and RD are chronic diseases associated with many complications, which can be systemic or organ-specific, including the musculoskeletal, endocrine, hematological, respiratory, and gastrointestinal systems, among others.^18^ Population-based studies examining the burden of mortality associated with AD are limited, so mortality data is underestimated.^19^

The present study represents a critical analysis of data on the causes of death based on autopsy results in patients with AD and RD, compared with data in Colombia and other South American countries. Iriya et al.^20^ previously analyzed 113 autopsies on patients with SLE in Brazil between 1981 and 1994. Our study reported 47 autopsy cases in patients with AD and RD. One fact to highlight is that despite the prevalence of AD in women, our study did not show this exacerbated relationship. There may be several reasons. Firstly, the nature of our study is retrospective, so it will have statistical biases. In addition, this study includes patients with gouty arthritis, a prevalent disease in men, which causes the female: male ratio to decrease due to our low sample size. There are other studies, such as those by Mendoza et al.,^6^ Diaz-Perez and Melo-Uribe,^7^ Bonilla Jassir et al.,^21^ and Braggion-Santos et al.,^22^ where they have done an extensive analysis of autopsies; however, no specific data on patients with AD and RD can be found in their records.

Infectious diseases were the main finding in our study, representing about 66% of the causes of death in patients who underwent autopsy. This finding is similar to autopsy studies previously conducted in other populations. Kitahama et al.^23^ performed a collection of 1225 patients with RA in Japan during two periods, 2000-2004 and 1985-1989. The leading cause of death in patients diagnosed with RA was infection (32.8%), followed by respiratory diseases (20.2%) and AA-amyloidosis (11,3%). Similarly, in the Brazilian study by Iriya et al.^20^ with patients with SLE, it turned out that infectious diseases were related to 57.5% of deaths, followed by lupus activity (33.62%).

Infections are a significant cause of mortality and morbidity in people with AD.^24^ The understanding of the relationship and interaction between AD and infection is not clear; however, it is known that exposure to pathogens is one of the environmental factors that can initiate or exacerbate AD.^25^ Another independent factor that carries a greater risk of infections is immunosuppressive treatment.^26^ In our study, almost all patients have been prescribed steroids (n=41/47, 87.23%). Patients with RA have twice the risk of acquiring an infection compared to the general population.^27^ In our study, 78.6% (n=11/14) of deaths in patients with RA and 63.6% (n=12/19) of patients with SLE had an infectious cause of death. Half of the cases of RA and a quarter of patients with SLE with infections were classified with OI, which can be defined as a generally progressive infection by a microorganism that has a limited pathogenic capacity (or none) under normal circumstances but which may cause severe disease due to the predisposing effect of another disease or its treatment. OI can be viral, bacterial (including mycobacteria), fungal or parasitic infections.^28^ Recently, a subgroup of patients included in our study with OI was described in detail in a case series. The most frequently reported infection was tuberculosis, followed by invasive fungal infections.^29^

Organ involvement (renal failure, liver failure, MODS) was the second cause of death in our study, which can be related to the inflammatory activity of AD in its different target organs, such as the kidney for patients with SLE (n = 8/19, 42%) and anti-GBM disease (n=2/2, 100%), as well as the liver for HA (n=2/3, 67%). Quadrelli et al.^30^ carried out an autopsy study in 90 patients with SLE, where it was possible to demonstrate, after carrying out histological studies, that the organs with the most inflammatory activity related to the disease were the kidneys (95.6%), the spleen (88.9%) and the lungs (87.8%). Similarly, in the patients with SLE in the study by Iriya et al.,^20^ the most affected organs were the brain, lungs, and heart.

Cardiovascular disease was the minority of causes of death in our group of patients (n=3, 6.38%), which is different from previously published reports. Even though autoimmune diseases carry a higher cardiovascular risk, the development of major cardiovascular events is also influenced by factors such as age (the median age of our patients is 39 years, which is considered relatively young) and additional comorbidities that are not reported in the autopsy reports of the patients in our study, in addition, our sample size was small. The study by Koivuniemi et al.,^31^ comprising 513 autopsies performed on RA patients, demonstrated that cardiovascular disease was the cause of death in 61% of cases, followed by RA-related activity (38%) and infections (27%). In recent years, it has been shown that patients with RA have higher mortality from cardiovascular disease than the general population, mainly due to a higher risk of developing metabolic disorders.^32^ Aubry et al.^33^ compared the histological features of coronary artery disease in RA patients and controls, showing that RA patients have less atherosclerosis but more significant evidence of inflammation and instability.

This study is of great importance since there has been little research about autopsies currently, being the study of AD or RD population even more unusual. This article is the first report on causes of death in patients with autoimmune and rheumatic diseases in Colombia.

However, this study had limitations in its conduct and analysis. First, due to its retrospective nature, it is impossible to give conclusions with statistical weight due to our small sample, and to obtain crucial clinical information such as dose and adherence to pharmacological management. Second, in patients diagnosed with pneumonia or septic shock without OI, the samples were not cultured because the tissue had already been in contact with formaldehyde in the pathology laboratory, making it impossible to perform; additionally, we do not have molecular tests in our institution. For this reason, this article is the impetus for the development of similar projects in the future.

## CONCLUSIONS

The autopsy is essential in health institutions to provide an accurate post-mortem diagnosis. This study focuses on determining the causes of death in patients with autoimmune and rheumatic diseases. Most of the deaths in our patients were related to an infectious etiology (opportunistic infections, pneumonia, septic shock). The cases caused by opportunistic organisms were of great relevance. Hence, the autopsy is essential in its diagnosis since, in most of these, the definitive diagnosis is given after histological analysis of the tissue.
